# Cuff Pressure in Pediatric Patients: From Monitoring to Clinical Practice

**DOI:** 10.7759/cureus.96956

**Published:** 2025-11-16

**Authors:** Joana Gomes, Sara Cabete, Luís Pedro, Tiago Miguel Cardoso, Sónia Duarte

**Affiliations:** 1 Clinic of Anesthesiology, Intensive Care, Emergency and Urgent Care Medicine, Unidade Local de Saúde de Santo António, Porto, PRT; 2 Clinic of Anesthesiology, Intensive Care, Emergency and Urgent Care Medicine, Unidade Local de Saúde de Santo António, Porto, PRT

**Keywords:** airway complications, airway management, cuff pressure, pediatric anesthesiology, pediatric surgery

## Abstract

Introduction: Monitoring of endotracheal tube cuff pressure (Pcuff), although recommended, is not mandatory in daily anesthetic practice. An adequate Pcuff is essential to ensure adequate ventilation, prevent potential microaspirations, and avoid airway complications. The goal of this clinical audit was to evaluate Pcuff after anesthetic induction and at the end of the procedure in pediatric patients undergoing general anesthesia with endotracheal intubation.

Methods: This prospective audit included all pediatric patients (<18 years) who underwent endotracheal intubation for elective and urgent surgeries during one month. After anesthetic induction, Pcuff values were measured objectively using a portable and calibrated cuff manometer and adjusted to the reference range (20-30 cmH_2_O) if outside this interval. A new evaluation was performed at the end of the surgical procedure. Pcuff values at those two time points were recorded, along with demographic, surgical, anesthetic, and intubation-related data.

Results: Fifty patients were included, with five excluded due to the absence of Pcuff measurements at the start or end of the procedure. After anesthetic induction, 37.8% (n=17) of the 45 patients had Pcuff within the reference range, while 28.9% (n=13) had Pcuff below 20 cmH_2_O, and 33.3% (n=15) exceeded 30 cmH_2_O. At the end of the procedure, 51.1% (n=23) of patients had Pcuff below 20 cmH_2_O, 2.2% (n=1) had Pcuff above 30 cmH_2_O and 46.7% (n=21) were within the 20-30 cmH2O range. There was a positive correlation between age and Pcuff at the end of surgery. Procedure duration and the type of tube did not appear to influence final cuff pressure.

Conclusion: More than 50% of the patients presented an inadequate Pcuff after anesthetic induction as well as at the end of the procedure. This study highlights that the routine implementation of objective and quantitative Pcuff measurements after induction and during the procedure could help maintain normal Pcuff values during anesthesia, potentially reducing complications related to pressure changes.

## Introduction

Current evidence supports the routine use of cuffed endotracheal tubes in the pediatric population [1]. In addition to selecting the appropriate tube size and insertion depth, one of the aspects that should not be overlooked when using cuffed tubes in the pediatric population is the cuff pressure (Pcuff), with the recommended range being 20-30 cmH_2_O [2]. Although the benefits of routine cuff pressure monitoring are well established, in the clinical setting, this practice remains underused [3].

The risks of out-of-range Pcuff are well documented, with underinflation of the cuff potentially leading to aspiration phenomena and compromising adequate ventilation and, conversely, overinflation causing sore throat, hoarseness and airway edema, compromising extubation due to the irritation and inflammation of the tracheal mucosa. Prolonged excessive cuff pressure can further compromise tracheal irrigation, leading to ischemic mucosa events that may predispose nerve injury, ulceration, stenosis, or, in severe cases, tracheal rupture [4-7].

Several subjective techniques for estimating Pcuff have been described, including manual palpation of the pilot balloon, the minimum leak test, the minimum occlusive volume method, and predetermined inflation volumes [3]. However, compared with objective methods of measuring the Pcuff, such as using a manometer, automated control devices or pressure-sensing syringes, the subjective methods may lead to significant inaccuracy and patient-to-patient variability [8,9]. In contrast, point-of-care manometer pressure measurement is a simple, reproducible and rapid technique that ensures adequate cuff pressure, thereby minimizing the risks of an out-of-range pressure and reassuring a safer procedure for the patient.

Although there is general knowledge about this issue, there are currently no universally accepted guidelines for routine cuff pressure monitoring in pediatric anesthesia. The present audit aims to alert to the inadequacy of cuff pressure when subjective methods are used and to highlight the importance of implementing objective measurements to monitor the cuff pressure, not only after intubation but also throughout the surgical procedure.

A resume of the audit (not the article) was presented as a poster on the Encontro Nacional de Internos de Anestesia, ENIA Congress, Portugal, on 11th October 2024.

## Materials and methods

This audit-based prospective observational study was conducted over a one-month period in the pediatric operating rooms of a Portuguese university hospital. As a clinical audit, approval was obtained from the institutional Quality Department (reference G.C. 005/2024), and informed consent was not required.

All consecutive pediatric patients (<18 years old) undergoing elective or urgent surgery under general anesthesia with tracheal intubation during the study period were included.

Endotracheal cuff pressure was measured using a Rusch® EndoTest™ cuff manometer (Teleflex, Wayne, USA) immediately after anesthetic induction and endotracheal intubation and measured again at the end of the surgery, prior to anesthetic emergence. Intubation was performed by an anesthesiologist or an anesthesiology resident. Cuff inflation was subsequently performed by the anesthesia nurse using a 5 or 10 mL syringe. The volume of air injected was determined according to the manufacturer’s recommendation for each specific tube size and brand, as stated on the tube packaging.

Whenever the initial Pcuff value was outside the recommended range, it was adjusted to values between 20 and 30 cmH_2_O. Pressures were categorized as <20 cmH₂O, 20-30 cmH₂O, or >30 cmH₂O, based on established pediatric airway safety limits.

Collected data included demographic, anesthetic and surgical data, as well as Pcuff after intubation, Pcuff at the end of the surgery, tube size, tube type and intubation method.

Data analysis was performed using IBM SPSS Statistics for Windows, Version 30 (Released 2024; IBM Corp., Armonk, New York, United States). Continuous variables were assessed for normality using the Shapiro-Wilk test. Categorical variables were expressed as frequencies and percentages.

Associations between continuous and ordinal variables were evaluated using Spearman’s rank correlation coefficient (ρ). Comparisons of continuous variables across three or more ordinal groups were performed with the Kruskal-Wallis test when normality assumptions were not met.

Associations between categorical variables were examined using Pearson’s chi-square test of independence or Fisher’s exact test (Freeman-Halton extension). 

All statistical tests were two-tailed, and a p-value < 0.05 was considered statistically significant.

## Results

A total of 50 patients were identified for the study. As five patients were excluded because of missing data (missed measurement at one of the points), 45 patients were included.

The study population had a mean age of 6.4 years (range: 11 days to 17 years). The distribution of age groups is presented in Table 1. The age groups are defined as follows: neonates (<28 days), infants (<1 year), children (1-12 years), and adolescents (13-18 years). The majority of patients were male (n = 28, 62.2%). According to the American Society of Anesthesiologists (ASA) Physical Status classification, 13 patients (28.9%) were classified as ASA I, 24 patients (53.3%) as ASA II, and eight patients (17.8%) as ASA III. Mean weight was 28.6 kg, with a range from 2.8 kg to 75 kg.

**Table 1:** Data on age group, surgical specialty, duration of procedure, type of anesthesia and type of endotracheal tube

Data collection			
Pediatric age group			
Neonates	1 (2.2%)		
Infants	5 (11.1%)		
Children	31 (68.9%)		
Adolescent	8 (17.8%)		
Surgical specialty			
ENT surgery	19 (42.4%)		
Pediatric surgery	17 (37.8%)		
Stomatology	3 (6.6%)		
Orthopedics	2 (4.4%)		
Neurosurgery	2 (4.4%)		
Urology	1 (2.2%)		
Plastic surgery	1 (2.2%)		
Duration of procedure			
<60 min	29 (64%)		
60-180 min	14 (31%)		
>180 min	2 (5%)		
Type of anesthesia			
General anesthesia	36 (80%)		
Combined anesthesia (general+caudal or epidural block)			9 (20%)
Type of endotracheal tube			
Oral RAE (AGT Rüsch^® ^Teleflex^TM^)	22 (48.9)		
Normal (Rüsch^®^ Safety Clear^® ^Teleflex^TM^)		11 (24.4)	
Microcuff (Microcuff *Avanos^TM^)		8 (17.8)	
Double Lumen (Rüsch^®^ Bronchopart^® ^Teleflex^TM^)			4 (8.9)

Information about age groups, surgical specialty, duration of procedure, type of anesthesia and type of endotracheal tube is displayed in Table 1. 

After anesthetic induction and before the start of surgery, cuff inflation was performed by the anesthesia nurse following the manufacturer’s instructions for the recommended inflation volume specified for each tube size and brand. At this initial assessment, only 37.8% of patients (n = 17) had cuff pressures within the recommended range (20-30 cmH₂O). Cuff pressure was then adjusted to the normal range.

At the end of the surgical procedure, only 46.7% of cases maintained an adequate cuff pressure. This indicates that even after manual correction, cuff pressure frequently drifted outside the recommended range during the course of anesthesia.

The data about Pcuff at the beginning and at the end of surgery are presented in detail in Table 2. 

**Table 2:** Pcuff after intubation and at the end of procedure Pcuff: Cuff pressure

Timing of measurement	Pcuff <20cmH_2_O	Normal Pcuff	Pcuff>30cmH_2_O
After intubation	13 (28.9%)	17 (37.8%)	15 (33.3%)
End of procedure	23 (51.1%)	21 (46.7%)	1 (2.2%)

For the statistical analysis, correlation and association tests were conducted to evaluate the potential impact of patient age, procedure duration, and endotracheal tube type on final cuff pressure values.

Age and Pcuff

A Spearman’s rank correlation analysis demonstrated a statistically significant moderate positive association between patient age and final endotracheal cuff pressure (ρ = 0.480, p < 0.001). Older children tended to exhibit higher cuff pressure values at the end of anesthesia compared with younger patients. Test statistics are reported in Table 3.

**Table 3:** Spearman's rank correlation Pcuff: Cuff pressure

Statistical Test	Variable Pair	N	Correlation Coefficient (ρ)	p-value	Interpretation
Spearman’s rank correlation	Age and Pcuff	45	ρ = 0.480	< 0.001	Moderate positive correlation (statistically significant)

When age was categorized into developmental age groups (neonates, infants, children and adolescents), the association between age group and final cuff pressure category was analyzed using the Pearson chi-square test. The overall association was not statistically significant (χ²(88) = 90.000, p = 0.421). However, the linear-by-linear association test revealed a significant positive trend (χ²(1) = 5.726, p = 0.017), indicating that higher cuff pressure values were indeed more frequent among older children. Test statistics are reported in Table 4.

**Table 4:** Test statistics using Pearson chi-square and linear-by-linear association Pcuff: Cuff pressure

Statistical Test	Variable Pair	N	Test Statistic	dF	p-value	Interpretation
Pearson chi-square	Age group and Pcuff	45	χ² = 90.000	88	0.421	No statistically significant association between age group and Pcuff
Linear-by-linear association	Age group and Pcuff	45	χ² = 5.726	1	0.017	Significant positive trend: higher cuff pressures more frequent among older children

Procedure duration

Procedure duration did not appear to influence final cuff pressure. Prior to analysis, the distribution of procedure duration was assessed using the Shapiro-Wilk test, which demonstrated a significant deviation from normality (W = 0.735, p < 0.001). A Kruskal-Wallis test revealed no statistically significant difference in procedure duration among the three final cuff pressure categories (<20, 20-30, and >30 cmH₂O) (H(2) = 2.405, p = 0.300). Mean rank values were comparable between the <20 cmH₂O (22.22) and 20-30 cmH₂O (22.90) groups, whereas the >30 cmH₂O group presented a higher mean rank (43.00), although this category included only one patient. Test statistics are reported in Tables 5, 6.

**Table 5:** Shapiro-Wilk analysis of normality (procedure duration)

Statistical Test	Variable Pair	N	Statistic (W)	p-value	Interpretation
Shapiro-Wilk	Procedure Duration	45	0.735	< 0.001	Distribution deviated significantly from normality

**Table 6:** Kruskal-Wallis procedure duration and Pcuff Pcuff: Cuff pressure

Statistical Test	Variable Pair	Test Statistic (H)	dF	p-value	Interpretation
Kruskal-Wallis	Procedure Duration and Pcuff	2.405	2	0.300	No statistically significant difference in the procedure duration among cuff pressure categories

Tube type

Similarly, the type of endotracheal tube was not significantly associated with final cuff pressure. Fisher’s exact test showed no significant relationship between tube type (normal, microcuff, RAE oral, or double-lumen) and final cuff pressure category (p = 0.200). The distribution of cuff pressure categories was similar across tube types, with most cases in each group exhibiting pressures within the 20-30 cmH₂O range. Test statistics are reported in Table 7. 

**Table 7:** Pearson chi-square/Fisher's exact test- tube type and Pcuff Although the chi-square test showed no significant relationship between the tube type and final cuff pressure, there was a need to adjust for small expected counts. Therefore, the Fisher–Freeman–Halton Exact Test was applied using Monte Carlo estimation, which confirmed the non-significant result (p = 0.200). Pcuff: Cuff pressure

Statistical Test	Variable Pair	Test Statistic	dF	p-value	Interpretation
Pearson Chi-square	Tube Type and Pcuff	χ² = 8.254	6	0.220	No statistically significant association between the tube type and final cuff pressure
Fisher–Freeman–Halton Exact Test (Monte Carlo)	Tube Type and Pcuff	-	-	0.200	Confirmed absence of significant association (adjusted for small expected counts)

## Discussion

This prospective audit revealed that, in our current practice, inadequate endotracheal Pcuff values after tracheal intubation were more frequent than pressures within the recommended range. In all cases, inflation of the cuff was performed by an experienced anesthesia nurse, inflating the predetermined volume of air proposed by the manufacturer. This method provided inadequate Pcuff in 62% of cases, requiring adjustment of cuff pressure to the normal range (20-30 cmH_2_O). These findings are comparable to the ones found by Sripadungkul [10], who tested if subjective inflation techniques (minimal occluding volume and stethoscope-guided technique) were reliable in the pediatric population and found that less than half of the cases achieved adequate cuff pressure, similar to the results of our audit.

Even after initial adjustment, fewer than half of the patients maintained adequate Pcuff values at the end of the surgical procedure. This observation may be related to exchanges in the patient positioning or with the surgery itself, since most of our procedures were in ENT surgery, with a shared airway with the surgeon, which might compromise the correct seal of the orotracheal tube cuff. This alerts to the need for repeated measurement throughout the procedure, in order to assure an ideal Pcuff pressure and hence minimize the risk of postextubation complications.

The moderate positive correlation between age and final cuff pressure (Tables 3, 4) suggests that increasing age is associated with higher intra-cuff pressures at the conclusion of anesthesia. This implies that younger children tend to have lower final cuff pressures and are at a bigger risk of cuff insufficiency towards the end of the surgery. This relationship may reflect anatomical and physiological differences among children, such as larger tracheal diameters requiring greater cuff inflation volumes, or variations in airway management techniques across age groups. Although moderate in strength, the correlation was statistically robust (p < 0.001), highlighting age as a relevant factor in cuff pressure monitoring and adjustment during pediatric anesthesia. Again, our findings in correlation between age and final cuff pressure are comparable to the ones reported by Sripadungkul et al. [10]

In contrast, the procedure duration did not significantly affect final cuff pressure values (Tables 5, 6). The absence of a meaningful association (H(2) = 2.405, p = 0.300) suggests that cuff pressure remains relatively stable throughout the surgical period and is not directly influenced by the length of anesthesia. Given the small number of cases with pressures above 30 cmH₂O, particularly a single observation in that category, these findings should be interpreted with caution. Also, two-thirds of the surgeries took less than 60 minutes, which can also influence the results obtained. Nevertheless, they indicate that intraoperative factors such as gas diffusion, positioning, and ventilation time may have a limited impact on cuff pressure changes when appropriate monitoring is maintained.

Likewise, no significant association was found between tube type and final cuff pressure (Table 7: χ²(6) = 8.254, p = 0.220; Fisher-Freeman-Halton p = 0.200). This finding suggests that, within this cohort, the configuration or material of the endotracheal tube did not substantially influence intra-cuff pressure outcomes. Previous research has reported that cuff pressure is primarily affected by the method of inflation, the precision of monitoring, and intraoperative ventilation variables, rather than by tube design alone [1,5,10]. However, the uneven distribution of tube types and small sample sizes in certain categories (e.g., microcuff and double-lumen tubes) limits the generalizability of this analysis. Future studies including larger and more homogeneous samples are warranted to determine whether specific tube designs may contribute to more consistent cuff pressure control and improved airway safety in pediatric populations.

This study has several limitations that should be acknowledged. The sample size was relatively small and drawn from a single center, which restricts the generalizability of the findings. Additionally, although the predominance of ENT surgeries provided a consistent procedural context, it may also have limited the applicability of the results to other surgical specialties. Moreover, the majority of procedures were of short duration (<60 minutes), which could have masked potential associations between prolonged anesthesia and intra-cuff pressure changes. These factors should be considered when interpreting the results, and future multicenter studies with larger sample sizes and longer surgical durations are needed to validate these findings and better characterize the temporal behavior of cuff pressure during pediatric anesthesia.

## Conclusions

In this audit, inadequate endotracheal Pcuff values, both immediately after intubation and at the end of surgery, were more prevalent than pressures within the recommended range, raising concern regarding the potential postoperative complications associated with suboptimal cuff pressure control. Statistical analysis demonstrated a moderate positive correlation between patient age and final Pcuff values, whereas neither procedure duration nor tube type showed a significant association with final cuff pressure.

Routine implementation of objective and quantitative Pcuff monitoring after induction and throughout anesthesia could help maintain pressures within the safe range and potentially reduce the risk of airway morbidity related to endotracheal intubation.
